# Trends in poverty risks among people with and without limiting-longstanding illness by employment status in Sweden, Denmark, and the United Kingdom during the current economic recession – a comparative study

**DOI:** 10.1186/1471-2458-13-925

**Published:** 2013-10-04

**Authors:** Johanna Falk, Daniel Bruce, Bo Burström, Karsten Thielen, Margaret Whitehead, Lotta Nylén

**Affiliations:** 1 Department of Public Health Sciences, Karolinska Institutet, Stockholm SE-171 77, Sweden; 2 Department of Public Health, University of Copenhagen, Copenhagen DK-1014, Denmark; 3 Department of Public Health and Policy, Institute of Psychology, Health and Society, University of Liverpool, Liverpool L69 3GL, UK

**Keywords:** Employment, Limiting-longstanding illness, Poverty risks, Welfare state

## Abstract

**Background:**

Previous studies have found higher employment rates and lower risk of relative poverty among people with chronic illness in the Nordic countries than in the rest of Europe. However, Nordic countries have not been immune to the general rise in poverty in many welfare states in recent decades. This study analysed the trends in poverty risks among a particularly vulnerable group in the labour market: people with limiting-longstanding illness (LLSI), examining the experience of those with and without employment, and compared to healthy people in employment in Sweden, Denmark and the United Kingdom.

**Methods:**

Cross-sectional survey data from EU-SILC (European Union Statistics on Income and Living Conditions) on people aged 25–64 years in Sweden, Denmark and the United Kingdom (UK) were analysed between 2005 and 2010. Age-standardised rates of poverty risks (<60% of national median equalised disposable income) were calculated. Odds ratios (ORs) of poverty risks were estimated using logistic regression.

**Results:**

In all three countries, non-employed people with LLSI had considerably higher prevalence of poverty risk than employed people with or without LLSI. Rates of poverty risk in the UK for non-employed people with LLSI were higher than in Sweden and Denmark. Over time, the rates of poverty risk for Swedish non-employed people with LLSI in 2005 (13.8% CI=9.7-17.8) had almost doubled by 2010 (26.5% CI=19.9-33.1). For both sexes, the inequalities in poverty risks between non-employed people with LLSI and healthy employed people were much higher in the UK than in Sweden and Denmark. Over time, however, the odds of poverty risk among British non-employed men and women with LLSI compared with their healthy employed counterparts declined. The opposite trend was seen for Swedish men: the odds of poverty risk for non-employed men with LLSI compared with healthy employed men increased from OR 2.8 (CIs=1.6-4.7) in 2005 to OR 5.3 (CIs=3.2-8.9) in 2010.

**Conclusions:**

The increasing poverty risks among the non-employed people with LLSI in Sweden over time are of concern from a health equity perspective. The role of recent Swedish social policy changes should be further investigated.

## Background

Developed welfare systems have a fundamental purpose to reduce poverty and improve living conditions among disadvantaged groups. Today growing numbers of working-age people are outside the labour market due to disease or disability [1]. Crucial contributing factors to this development are the retrenchment of the welfare state, globalisation, deindustrialisation and the post-industrial labour market changes [2-5]. In addition, the previous economic recession of the 1990s had adverse consequences on employment among groups with poor health in Sweden [6]. Further, studies have shown social differentials in health-related employment consequences in that certain groups, such as manual workers and women with LLSI are more susceptible to loss of employment [7-11]. Moreover, poor health striking in young adulthood seems to result in more adverse employment consequences compared to a similar situation later in life, particularly among people with low educational attainment [12].

Living conditions are affected not only by the opportunities in the labour market for people with LLSI, but also by the adequacy of social protection. Two main characteristics of the Nordic countries are high levels of employment and low poverty rates [13]. Additionally, these countries, including Sweden and Denmark, are also better at keeping people with disease and disability in employment compared to welfare systems such as the UK [14,15], possibly due to greater investment in active labour market policies [16]. Also, studies have found smaller social differentials in employment consequences of poor health among people in Sweden than in Britain [17,18].

Poverty reflects living conditions in terms of standard of living, and the lack of economic resources [19]. Relative poverty (measured as less than 60% of national median disposable income) captures people’s resources and ability to participate in the society in which they live, relative to others [20]. Poverty can have detrimental effects on health as it impairs living conditions in material, social and psychological aspects [21]. The importance of preventing poverty resulting from unemployment has been emphasized, as unemployment is part of the process of social exclusion [22]. Poverty is a prioritised issue in the European Union, and used as one indicator of social exclusion measured as at-risk-of-poverty, defined as disposable household income below 60 percent of the national median income [23].

Cross-national research has found that income from paid employment has become more important since the mid-1990s as a protective factor against poverty [24]. Further, increasing poverty risks have been seen in all modern welfare systems in the past decades, including the Nordic countries [24]. A study on labour market and social security in European countries from mid-1990s to late 2000s also shows convergence in poverty risks between the Nordic countries and the others, although the risks were still considerably lower than the average for the European Union countries and for the UK in particular [25].

In relation to people with limiting-longstanding illness (LLSI), risks of poverty in Nordic countries are expected to be lower, as these countries have relatively high levels of social protection. On the other hand, the recession and major policy changes, particularly striking in Sweden during this period, may have altered the longstanding pattern of poverty risks in different European countries, especially for groups in vulnerable positions in the labour market, such as those with LLSI and low socioeconomic status. This study aimed to analyse trends in poverty risks for people in different health/employment categories before and during the current economic recession in three contrasting European systems: Sweden, Denmark and the UK. The three countries were selected for comparison because all three experienced recession over the study period of 2005 to 2010, but they differed in their welfare system type, based on social protection and labour market policies of particular relevance to people of working age with LLSI. Sweden is an example of a country with high social protection and low labour market flexibility (one of the most highly regulated labour markets in Europe); the UK is an example of a country characterised by low social protection and high labour market flexibility; while Denmark is the quintessential ‘flexicurity’ country, combining high social protection with high labour market flexibility, including in relation to disabled workers [13,26]. It is possible, therefore, that the impact of recession on poverty risks and on employment may differ in these three welfare system policy contexts. Moreover, the adequacy and entitlements of different social protection systems have been subject to major policy changes. The recent Swedish social policy changes, for example, include changes in the tax system benefitting those in employment; differentiated unemployment insurance with higher fees leading to lower enrolment; and the introduction of a maximum period for receiving sickness benefit. These changes make it important to study how people with LLSI outside the labour market in Sweden fare in terms of poverty risks compared other countries. Corresponding policy changes on the scale experienced in Sweden have not been seen in Denmark or the UK.

## Methods

### Data

The study is based on annual cross-sectional survey data for the years 2005 to 2010 from the European Union Statistics on Income and Living Conditions survey (EU-SILC). The survey includes measures of income, labour market position, education and health and covers all European Union countries, except Malta, as well as Norway and Iceland [27]. In the EU-SILC cross-sectional survey data for selected countries, respondents may remain in the data for four consecutive years, as only a quarter of the sample consists of new respondents each year and one quarter is excluded, due to a four-year rotational design [28]. Thus, pooling of annual data was not possible. However, using each sample separately avoids this problem. The sample of 2005 and 2010, respectively, consists of independent individuals.

### Study population

The annual study population, aged 25-64 years, used for the time trend analyses between 2005 and 2010, ranged from 3 601 to 4 403 respondents in Sweden, from 3 558 to 3 701 respondents in Denmark and from 7 757 to 10 217 respondents in the UK. The study populations for the logistic regressions in 2005/2010 were 3 602/4 029 respondents in Sweden, 3 701/4 029 respondents in Denmark and 10 217/7 757 respondents in the UK.

In this study, three groups were formed, stratified by employment and health status. Healthy non-employed people were excluded due to the heterogeneous composition of this group between countries. In Sweden and Denmark, this group mainly consisted of unemployed people, but in the UK it also consisted of a sizable proportion describing themselves as ‘looking after the home’ (predominantly women). The non-response rate varied between 20-30 per cent [27,28].

### Measures

Employment was measured by self-defined current economic status, and dichotomized into employed (working full-/part-time, self-employed), and non-employed (unemployed, pupil, student, further training, unpaid work experience, in retirement or in early retirement/given up business, permanently disabled or/and unfit to work, fulfilling domestic tasks and care responsibilities, other inactivity). Only one category could be selected by respondents. People in military service were excluded. Health status was assessed by two questions: *Do you suffer from a longstanding illness/condition?*; *Does your illness/condition limit your activities?* People who answered ‘yes’ to both question were categorized as having limiting-longstanding illness (LLSI), and the others as healthy. Information on highest attained educational level was based on ISCED 97 (International Standard Classification of Education) and categorized into three levels: low education (0-2 low/lower secondary), intermediate education (3-4 medium/upper secondary completed) and high education (5-6 high/tertiary completed). This variable was dichotomised into low educational level yes/no. The outcome measure was being at-risk-of-poverty, defined as having an equivalised disposable income after social transfers below 60 per cent of the median national equivalised disposable income, according to the EU definition [23]. Income data were taken from registers in Sweden and Denmark, and from self-reported data in the UK. In the subsequent text, being at-risk-of-poverty is referred to as poverty risks. Age was categorized into two groups: 25-44 yrs, 45-64 yrs.

### Statistical analysis

In each country, three population categories were formed (healthy/employed; LLSI/employed; LLSI/non-employed) and distributions of the older age group (45-64 yrs), women, low educational level and prevalence of poverty were calculated. For each country and year, these proportions were compared between non-employed people with LLSI and the two other health/employment categories, respectively, using chi-square test.

In order to study differentials in employment between healthy people and those with LLSI in each country, age-standardised employment rates were calculated. Annual age-standardised prevalence of poverty risks was estimated for a six-year period between 2005 and 2010 to assess possible trends. The number of observations was not sufficient for sex-stratified analyses. Age-standardised prevalence of poverty by educational level was estimated, pooling the samples of 2005 and 2010 to increase statistical power. The European standard population was used in the age-standardized analyses, to increase the comparability between the population categories in the three countries [29].

Logistic regression models were fitted to estimate the odds ratio (OR) of poverty risks, in 2005 and 2010, separately. The aim was to study the relative inequalities in risk of poverty between people (men and women combined) in the three health/employment categories, and to identify possible changes in inequalities over the study period. An interaction term of employment status, health status and country was added in order to study variation in poverty risks between health/employment categories in each country. Firstly, we stratified the regression analyses by country, and calculated ORs for poverty risk for each of the three health/employment categories, adjusted for age, age and sex, age, sex and educational level. The reference group was people in the ‘healthy employed’ category in each country. Secondly, we included all countries in the analysis, using healthy employed people in Sweden as the reference category, as we expected this category to have the lowest poverty risk. The models were stratified by sex, and adjusted for age, and age and educational level. Evaluation of the models was made by comparing predicted probability of poverty risk to estimated probability. The analyses were conducted with the SAS software package 9.3.

### Ethical approval

The study was approved by Stockholm Regional Ethical Review Board (2010/2052-31/5).

## Results

Characteristics of the three health/employment categories by country in 2005 and 2010 are presented in Table 1. The chi-square test indicated that the poverty risk was significantly higher among non-employed people with LLSI compared to the other two health/employment categories, in all three countries and at both time points. The proportion of women was significantly higher among non-employed people with LLSI compared to healthy employed people in all three countries, but was only statistically significantly higher than for employed people with LLSI in the UK. The proportion of people with low educational level was statistically significantly higher among non-employed people with LLSI than among healthy employed people in all three countries in both time periods, but was only statistically significantly higher than for employed people with LLSI in 2010 in Sweden and Denmark, and for both time periods in the UK. Age-standardised employment rates among people with LLSI in 2005 and 2010 were statistically significantly higher in Sweden and Denmark compared to the UK (Table 2).

**Table 1:** Socio-demographic characteristics of the three health/employment categories, by country and year

	**% 45-64 yrs**	**% women**	**% low educational level**	**% at-risk-of-poverty**	**Total N**
**Sweden 2005**					
Non-employed with LLSI	70.7^ab^	58.0^b^	28.1^b^	12.1^ab^	362
Employed with LLSI	59.2	55.8	15.0	4.2	448
Healthy employed	47.5	47.6	10.5	4.4	2 791
**Sweden 2010**					
Non-employed with LLSI	74.6^ab^	63.6^b^	24.6^ab^	23.3^ab^	236
Employed with LLSI	62.5	63.7	10.7	6.7	355
Healthy employed	49.0	49.2	8.3	6.3	3 438
**Denmark 2005**					
Non-employed with LLSI	73.0^ab^	66.7^b^	39.2^b^	9.7^ab^	237
Employed with LLSI	56.1	63.0	21.2	5.2	289
Healthy employed	47.6	47.8	16.6	2.9	3 175
**Denmark 2010**					
Non-employed with LLSI	71.9^ab^	63.9^b^	37.4^ab^	13.3^ab^	263
Employed with LLSI	62.8	56.2	16.9	5.9	306
Healthy employed	56.0	51.6	13.6	4.6	2 797
**UK 2005**					
Non-employed with LLSI	72.8^ab^	58.0^ab^	46.1^ab^	36.7^ab^	1 278
Employed with LLSI	56.2	51.0	15.4	10.4	1 030
Healthy employed	44.4	48.6	13.9	6.6	7 909
**UK 2010**					
Non-employed with LLSI	75.8^ab^	56.7^ab^	40.2^ab^	33.8^ab^	950
Employed with LLSI	64.6	54.1		7.9	695
Healthy employed	50.7	48.6	11.5	6.9	6 112

^a^Statistically significant difference (p < 0.05) between non-employed people with LLSI and employed people with LLSI.

^b^Statistically significant difference (p < 0.05) between non-employed people with LLSI and healthy employed people.

**Table 2:** Age-standardised employment rates by health status in each country, by year, percentages (95% confidence intervals)

	**Sweden**		**Denmark**		**UK**	
	**2005**	**2010**	**2005**	**2010**	**2005**	**2010**
**Health status**	% (95% CI)	% (95% CI)	% (95% CI)	% (95% CI)	% (95% CI)	% (95% CI)
LLSI	56.5 (53.0-60.0)	62.2 (58.2-66.3)	57.3 (53.0-61.5)	55.0 (50.7-59.2)	46.9 (44.8-49.0)	44.8 (42.2-47.4)
Healthy	87.8 (86.7-89.0)	87.3 (86.3-88.3)	83.9 (82.8-85.1)	84.1 (82.8-85.3)	79.0 (78.2-79.8)	80.2 (79.3-81.1)

**Figure 1:**
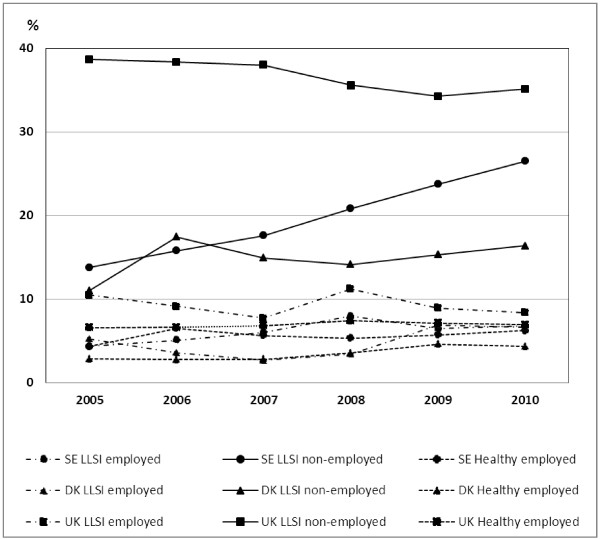
Annual age-standardised prevalence (%) of poverty risk among the three health/employment categories (men and women combined) in Sweden, Denmark, and the UK from 2005 to 2010.

The prevalence of poverty during the period 2005-2010 was considerably higher among non-employed people with LLSI, compared to the other two categories in each country, except in Denmark in 2005 (Figure 1). The prevalence was highest and relative inequalities greatest between the health/employment categories in the UK. A statistically significant increase in prevalence of poverty risk was seen among non-employed people with LLSI in Sweden between 2005 and 2010 (prevalence of poverty risk increased from 13.8 per cent (CI=9.7-17.8) in 2005 to 26.5 per cent (CI=19.9-33) in 2010 (Figure 1). A smaller, non-significant increase was seen in Denmark. In the UK, the prevalence remained on a higher level than Sweden and Denmark, but decreased slightly over time, although not statistically significantly (Figure 1).

Stratified analyses by educational level of the pooled sample of years 2005 and 2010 showed that poverty risks increased with decreasing level of education (data not shown). Poverty risks among non-employed people with LLSI were statistically significantly higher than for healthy employed people, at all educational levels, and were particularly high in the UK. In 2005, poverty risks among non-employed people with LLSI were highest in the UK and lowest in Sweden (Table 3). The corresponding analyses in 2010 found highest poverty risks for non-employed people with LLSI in the UK, but lowest in Denmark. Other adjustments did not alter the results significantly (models 2 and 3). The relative inequalities in poverty risks between non-employed with LLSI and healthy employed categories increased from 2005 to 2010 in Sweden, decreased slightly in the UK, and remained unchanged in Denmark (Table 3).

**Table 3:** Poverty risks for the three health/employment categories by country and year, odds ratio (OR) with 95% confidence interval (CI) adjusted for: age (model 1); age and gender (model 2); age, gender and educational level (model 3)

	**2005**						**2010**					
	**Model 1**		**Model 2**		**Model 3**		**Model 1**		**Model 2**		**Model 3**	
	**OR**	**95% Cl**	**OR**	**95% Cl**	**OR**	**95% Cl**	**OR**	**95% Cl**	**OR**	**95% Cl**	**OR**	**95% Cl**
**Sweden**												
Healthy employed (ref)	1.0		1.0		1.0		1.0		1.0		1.0	
Employed with LLSI	1.0	(0.6-1.7)	1.1	(0.7-1.8)	1.2	(0.7-1.8)	1.2	(0.8-1.8)	1.2	(0.8-1.9)	1.2	(0.8-1.8)
Non-employed with LLSI	3.6	(2.4-5.2)	3.8	(2.6-5.5)	3.6	(2.5-5.4)	5.3	(3.8-7.5)	5.5	(3.9-7.8)	4.9	(3.5-7.0)
**Denmark**												
Healthy employed (ref)	1.0		1.0		1.0		1.0		1.0		1.0	
Employed with LLSI	1.9	(1.1-3.4)	1.9	(1.1-3.4)	1.9	(1.1-3.4)	1.5	(0.9-2.4)	1.5	(0.9-2.5)	1.6	(0.9-2.6)
Non-employed with LLSI	4.2	(2.6-6.9)	4.2	(2.6-7.0)	3.8	(2.3-6.4)	3.8	(2.5-5.7)	3.9	(2.6-5.9)	4.0	(2.6-6.1)
**UK**												
Healthy employed (ref)	1.0		1.0		1.0		1.0		1.0		1.0	
Employed with LLSI	1.7	(1.4-2.1)	1.7	(1.4-2.1)	1.7	(1.3-2.1)	1.2	(0.9-1.6)	1.2	(0.9-1.6)	1.2	(0.9-1.6)
Non-employed with LLSI	8.7	(7.5-10.1)	9.0	(7.7-10.4)	7.2	(6.1-8.4)	7.1	(6.0-8.4)	7.2	(6.0-8.5)	6.1	(5.0-7.3)

Reference group, healthy employed people in each country.

In the regression analyses of poverty risks stratified by sex (Table 4), odds ratios adjusted for age (model 1) for both men and women who were non-employed with LLSI were statistically significantly higher than for men and women in the healthy employed category in all three countries and both time periods. These statistically significant higher odds of poverty for men and women in the non-employed with LLSI category remained after adjustments for educational level, for all three countries in 2005 and 2010, except for Danish non-employed women with LLSI in 2005 (Table 4, model 2).

**Table 4:** Poverty risks stratified by sex in the three health/employment categories, by country and year, odds ratio (OR) with 95% confidence interval (CI) adjusted for: age (model 1); age and educational level (model 2)

		**2005**				**2010**			
		**Model 1**		**Model 2**		**Model 1**		**Model 2**	
		**OR**	**95% Cl**	**OR**	**95% Cl**	**OR**	**95% Cl**	**OR**	**95% Cl**
**Men**									
**Sweden**									
	Healthy employed (ref)	1.0		1.0		1.0		1.0	
	Employed with LLSI	1.1	(0.6-2.1)	1.1	(0.5-2.0)	1.3	(0.7-2.5)	1.2	(0.6-2.4)
	Non-employed with LLSI	2.8	(1.6-4.7)	2.4	(1.4-4.2)	5.3	(3.2-8.9)	4.9	(2.9-8.3)
**Denmark**									
	Healthy employed	0.5	(0.3-0.7)	0.5	(0.3-0.7)	0.7	(0.5-1.0)	0.7	(0.5-0.9)
	Employed with LLSI	1.1	(0.5-2.5)	1.1	(0.4-2.5)	1.0	(0.5-2.1)	1.0	(0.5-2.0)
	Non-employed with LLSI	3.2	(1.6-6.3)	2.9	(1.5-5.8)	2.3	(1.3-4.3)	2.1	(1.1-3.9)
**UK**									
	Healthy employed	1.3	(1.0-1.7)	1.3	(1.0-1.3)	1.0	(0.8-1.3)	1.0	(0.8-1.3)
	Employed with LLSI	2.4	(1.7-3.5)	2.5	(1.7-2.5)	1.3	(0.8-2.0)	1.3	(0.8-2.1)
	Non-employed with LLSI	14.1	(10.5-18.8)	12.2	(9.1-16.5)	8.7	(6.6-11.5)	7.4	(5.5-10.0)
**Women**									
**Sweden**									
	Healthy, employed (ref)	1.0		1.0		1.0		1.0	
	Employed with LLSI	1.0	(0.5-2.1)	1.0	(0.4-2.0)	1.1	(0.6-1.9)	1.0	(0.5-1.8)
	Non-employed with LLSI	4.2	(2.5-7.0)	3.6	(2.1-6.0)	4.9	(3.1-7.6)	4.1	(2.6-6.5)
**Denmark**									
	Healthy employed	0.9	(0.6-1.4)	0.9	(0.6-1.3)	0.6	(0.4-0.9)	0.6	(0.4-0.8)
	Employed with LLSI	1.6	(0.7-3.2)	1.4	(0.7-2.9)	0.9	(0.5-1.9)	0.9	(0.4-1.8)
	Non-employed with LLSI	2.6	(1.3-4.9)	1.8	(0.9-3.6)	2.6	(1.6-4.3)	2.2	(1.3-3.6)
**UK**									
	Healthy employed	1.9	(1.4-2.7)	1.9	(1.3-2.6)	1.2	(0.9-1.5)	1.1	(0.9-1.5)
	Employed with LLSI	2.9	(1.9-4.5)	2.8	(1.8-3.7)	1.4	(0.9-2.2)	1.3	(0.9-2.1)
	Non-employed with LLSI	15.4	(10.9-21.6)	11.9	(8.4-16.8)	8.2	(6.2-10.8)	6.7	(5.0-9.0)

Reference group, healthy employed Swedes.

For both sexes and both time periods, the inequalities in poverty risk between non-employed people with LLSI and healthy employed people were much higher in the UK than in Sweden and Denmark (Table 4, model 1), a pattern that remained after adjustments for educational level (Table 4, model 2). Over time, however, the odds of poverty among British non-employed men and women with LLSI compared with their healthy employed counterparts declined. The opposite trend was seen for Swedish men: the odds of poverty risk for non-employed men with LLSI compared with healthy employed men increased from OR 2.8 (CIs=1.6-4.7) in 2005 to OR 5.3 (CIs 3.2-8.9) in 2010. There was no corresponding increase in the odds of poverty risk for Swedish non-employed women with LLSI compared with their healthy employed counterparts.

## Discussion

Non-employed people with LLSI had higher poverty risks compared to the employed, with or without LLSI, among both men and women, but the rates were lower in Sweden and Denmark than in the UK. Poverty risks were inversely related to the level of education, and differentials between the educational groups were largest in the UK, although they increased in Sweden over time. Further, people with LLSI in Sweden and Denmark had higher employment rates and lower poverty risks, compared to the corresponding category in the UK. The differentials between countries may partly reflect the more comprehensive social and labour market policies in the Nordic countries [14-16] which seem to promote employment and protect against poverty in vulnerable groups.

We found a considerable increase in the prevalence of poverty among Swedish non-employed people with LLSI during the period 2005 to 2010. This finding corresponds with recent Swedish data on increasing rates of poverty risks in the total population, from less than 10 per cent in 2005, to almost 13 per cent five years later [30]. These increasing poverty risks may reflect different macro-economic changes in recent years. Firstly, it may reflect increasing income inequalities in Sweden [31,32], and more specifically between 2005 and 2010, when income levels increased for all, except for the lowest income quintile [30]. The changes in the Swedish tax system in 2006 and onwards have primarily benefitted those in employment. Secondly, the increasing poverty risk among those outside the labour market is also indicative of the decreasing real value of social transfers and benefits, which has occurred over a longer time in Sweden [33]. The proportions of unemployed people covered by unemployment insurance benefit have also decreased from 70 per cent in 2004 to 55 per cent in 2008 [34]. In addition, Sweden has had a greater decline in unemployment benefit generosity than Denmark and the UK between 2005 and 2010 [35]. Lastly, the proportion on social benefit has increased in 2010 among people who reached the maximum duration of sickness insurance 2009, according to recent data [36].

Although income inequalities also increased in Denmark during the 2000s [30], the increasing poverty risks were much smaller than in Sweden, according to our study, which we could speculate are due to unchanged social benefit levels, and new active employment measures introduced in Denmark during the study period.

We also found a cross-over in the odds of poverty among the non-employed with LLSI in Swedish men and women over time: in 2005 the odds of poverty risk were higher for Swedish women than Swedish men for non-employed people with LLSI compared to their healthy employed counterparts. By 2010, the odds of poverty risk for the same category of Swedish men had risen, and overtaken those of Swedish women, for whom the odds had remained fairly constant. This resulted in similar relative odds of poverty risk between the categories among men and among women in 2010. One contributing factor may be the increased need for income support among Swedes who reach the maximum period for receiving sickness benefit, observed particular among men [36].

### Strengths and limitations

The main advantage of the cross-sectional EU-SILC data was the wide range of variables concerned with living conditions. The survey is a major source for comparative statistics in the EU, relevant for welfare state research, and specifically aimed at studying poverty risks in different populations. Due to the cross-sectional design of the survey, no conclusions on causality could be made. The national questionnaires are harmonised but limitations of comparability, may still exist due to diverse design and implementation of the survey in each country [37]. On the other hand, the interpretation of the survey information between the three countries is still valid, as we use a relative outcome measure. Also, the small sample size of the sub-groups was one limitation of the study. This caused statistical power problems which limited analyses stratified by sex and educational level, and gave wide confidence intervals. Each country in the EU-SILC survey uses statistical methods to compensate for underrepresented groups [28]. Still, underestimation of poverty risks among the worst off is likely, due to difficulties in reaching disadvantaged groups to participate in surveys [38]. The underestimation may limit the possibility to generalize the results to the whole population in each country. The measure of poverty risks is based on income data, which are taken from registers in Sweden and Denmark, and from self-reported data in the UK. This possible systematic bias between countries, however, occurs both in 2005 and 2010, and would not affect the interpretation of the changes in the differentials we found over time. limiting-longstanding illness was the only measure of health used in this study. It is considered to cover both chronic and severe diseases [39]. The measurement of educational level has good coverage in the EU-SILC survey, but is a crude measure of social position and misclassification may have occurred, due to the large heterogeneous groups [40].

## Conclusion

Our study shows higher poverty risks among non-employed people with LLSI than among those employed, and more pronounced differentials in the UK, than in Sweden and Denmark, have persisted from 2005 to 2010. What did change, however, is the considerable increase in poverty risk among Swedish non-employed people with LLSI between the two time points, a trend that was not seen in the UK or in Denmark, even though all three countries experienced recession during that period. This causes concern from a health equity perspective. The role of recent Swedish social policy changes need to be further investigated.

## Competing interests

The authors declare that they have no competing interests.

## Authors’ contributions

JF prepared and carried out statistical analyses and interpretation of the data, and drafted the manuscript under supervision. DB helped to plan, perform and interpret the statistical analyses and draft the manuscript. KT helped to draft the manuscript. MW contributed to the interpretation of results and helped to draft the manuscript. BB and LN, supervisors, acquired data, conceived of the study, participated in the design of the study, and helped to interpret the statistical analyses and draft the manuscript. All authors read and approved the final manuscript.

## Pre-publication history

The pre-publication history for this paper can be accessed here:

http://www.biomedcentral.com/1471-2458/13/925/prepub
